# Growth rate and nutrient limitation as key drivers of extracellular quorum sensing signal molecule accumulation in *Pseudomonas aeruginosa*

**DOI:** 10.1099/mic.0.001316

**Published:** 2023-04-05

**Authors:** Jean-Frédéric Dubern, Nigel Halliday, Miguel Cámara, Klaus Winzer, David A. Barrett, Kim R. Hardie, Paul Williams

**Affiliations:** ^1^​ National Biofilms Innovation Centre, Biodiscovery Institute, School of Life Sciences, University of Nottingham, Nottingham NG7 2RD, UK; ^2^​ Centre for Analytical Bioscience, Advanced Materials and Healthcare Technology Division, School of Pharmacy, University of Nottingham, Nottingham NG7 2RD, UK

**Keywords:** *Pseudomonas aeruginosa*, quorum sensing, continuous culture, *N*-acyl-homoserine lactone, 2-alkyl-4-quinolone, PQS, growth rate, population density, nutrient limitation, mass spectrometry

## Abstract

In *Pseudomonas aeruginosa *, quorum sensing (QS) depends on an interconnected regulatory hierarchy involving the Las, Rhl and Pq*s* systems, which are collectively responsible for the co-ordinated synthesis of a diverse repertoire of *N*-acylhomoserine lactones (AHLs) and 2-alkyl-4-quinolones (AQs). Apparent population density-dependent phenomena such as QS may, however, be due to growth rate and/or nutrient exhaustion in batch culture. Using continuous culture, we show that growth rate and population density independently modulate the accumulation of AHLs and AQs such that the highest concentrations are observed at a slow growth rate and high population density. Carbon source (notably succinate), nutrient limitation (C, N, Fe, Mg) or growth at 25 °C generally reduces AHL and AQ levels, except for P and S limitation, which result in substantially higher concentrations of AQs, particularly AQ *N*-oxides, despite the lower population densities achieved. Principal component analysis indicates that ~26 % variation is due to nutrient limitation and a further 30 % is due to growth rate. The formation of *N*-(3-oxododecanoyl)-l-homoserine lactone (3OC12-HSL) turnover products such as the ring opened form and tetramic acid varies with the limiting nutrient limitation and anaerobiosis. Differential ratios of *N*-butanoyl-homoserine lactone (C4-HSL), 3OC12-HSL and the AQs as a function of growth environment are clearly apparent. Inactivation of QS by mutation of three key genes required for QS signal synthesis (*lasI*, *rhlI* and *pqsA*) substantially increases the concentrations of key substrates from the activated methyl cycle and aromatic amino acid biosynthesis, as well as ATP levels, highlighting the energetic drain that AHL and AQ synthesis and hence QS impose on *P. aeruginosa *.

## Introduction

Bacteria coordinate population-dependent activities by producing and sensing diffusible signal molecules via quorum sensing (QS), which facilitates social behaviour in diverse species [1, 2]. As bacterial populations expand, the extracellular QS signal molecule (QSSM) concentration rises. Once a threshold concentration has been attained, a sensor kinase or response regulator protein is activated (or repressed), so controlling the expression of QS-dependent genes [1, 2]. The size of the quorum is not fixed but varies according to the relative rates of QSSM production, turnover and spatial diffusion [3]. QS systems do not, however, function in isolation, but are embedded within sophisticated regulatory hierarchies and modulated by environmental parameters such as nutrient availability, oxygen tension and temperature [4, 5]. Furthermore, QS can fine tune gene expression in response to graded environmental changes without a quorum being attained through shifts in the proportion of bacterial cells responding and the intensity of their responses [6].

**Fig. 1.**
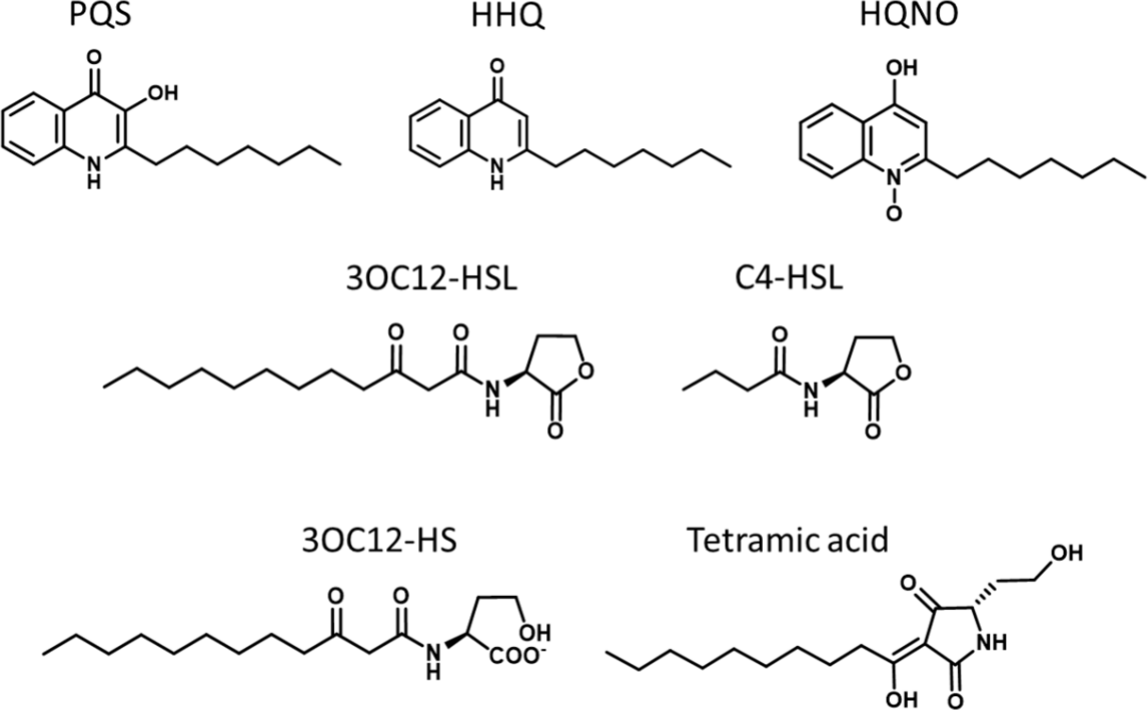
Structures of *P. aeruginosa* C4-HSL, 3OC12-HSL, PQS, HHQ and HQNO plus 3OC12-HS (open ring form) and tetramic acid.

In *Pseudomonas aeruginosa,* QS involves an interconnected hierarchical network involving the Las, Rhl and PQS systems that employ *N*-acylhomoserine lactone (AHL) or 2-alkyl-4-quinolone (AQ) signal molecules [4, 5] (Fig. 1). All three *P. aeruginosa* QS systems contain auto-induction loops in which activation of a dedicated transcriptional regulator by the cognate QS signal induces expression of the target synthase such that QS signal molecule biosynthesis can be rapidly amplified. Furthermore, the Las system, which is usually at the top of the QS hierarchy, regulates both the Rhl and Pqs systems such that inactivation of Las impacts on QSSM production by all three [4, 5]. However, under certain growth conditions and in some *P. aeruginosa* strains, the QS hierarchy is flexible [7–9].

The major AHLs (Fig. 1) synthesized via LasI and RhlI are *N*-(3-oxododecanoyl)-l-homoserine lactone (3OC12-HSL) and *N*-butanoyl-l-homoserine lactone (C4-HSL), respectively, which activate their transcriptional activators, LasR and RhlR [4, 5]. Concentrations ranging from 5 to 31 µM for C4-HSL and 0.5–15 µM for 3OC12-HSL in cell-free culture supernatants have been reported [10], while EC_50_s ranging from 1 to 8 μM for RhlR/C4-HSL and 10-139 nM for LasR/3OC12-HSL have been determined for their activation [11–13]. RhlI and LasI are also responsible for the biosynthesis of a range of minor AHLs with different acyl chain lengths and 3-position substituents [14]. The function of these is not clear, although some are weaker activators of LasR and RhlR than 3OC12-HSL and C4-HSL, while others have been reported to activate the orphan *P. aeruginosa* LuxR homologue QscR, which shows a more relaxed specificity for AHLs [15]. LuxR homologue-independent responses to AHLs have also been described [16].

*P. aeruginosa* also produces diverse AQs [14, 17], which possess antimicrobial, cytotoxic, iron chelating, immune modulatory and QS signalling activities [4, 5, 18]. These are characterized by the presence or absence of a 3-hydroxyl group, variations in the length of the 2-alkylside chain or *N*-oxidation [14, 17]. With respect to the AQs, the major QS signals are the *Pseudomonas* quinolone signal [PQS; 2-heptyl-3-hydroxy-4(1 *H*)-quinolone; Fig. 1] and its immediate biosynthetic precursor, 2-heptyl-4-hydroxyquinoline (HHQ; Fig. 1). Their synthesis depends on the *pqsABCDE* operon, which is under the positive control of the transcriptional regulator, PqsR (MvfR) [19, 20]. HHQ is oxidized to PQS via the action of mono-oxygenase PqsH [21]. Biosynthesis of respiration inhibiting antibacterial AQ *N*-oxides such as 2-heptyl-4-hydroxyquinoline *N*-oxide (HQNO) (Fig. 1), which do not function as signal molecules [20], requires the *pqsABCDE* operon plus the mono-oxygenase PqsL [22]. In *P. aeruginosa,* HQNO induces cell death and autolysis, promoting biofilm formation and antibiotic tolerance [23].

The major AQs are produced in micromolar concentrations in lysogeny broth (LB) medium in batch cultures [14]. PqsR is activated by both PQS and HHQ with EC_50_s of 1.8 and 0.8 µM, respectively, and also by the C9 alkyl chain congeners, 2-heptyl-4-hydroxyquinoline (NHQ) and 2-heptyl-3-hydroxy-4(1 *H*)-quinolone (C9-PQS), which have similar EC_50_s [20]. Numerous genes involved in virulence, secondary metabolism, swarming and biofilm development are regulated via the Las, Rhl and Pqs systems. In batch cultures, deletion of both *lasI* and *rhlI* results in ‘metabolic rewiring’ and perturbations of primary metabolic pathways, including, for example, the tricarboxylic acid (TCA) cycle, amino acid and fatty acid metabolism [24].

Biosynthesis of QSSMs depends on the availability of substrates from primary metabolism. These are mainly obtained from fatty acid biosynthesis and the activated methyl cycle (AMC) for the AHLs via the supply the acyl chains (via acyl-ACPs) and the homoserine lactone moiety [derived from *S*-adenosyl methionine (SAM)], respectively [25]. The AQs are synthesized from fatty acyl-CoA intermediates and anthranilate produced from chorismate via dedicated anthranilate synthases (TrpEG and PhnAB) or via tryptophan degradation [26]. Given that AQ production is partially dependent on the Las system [4], reductions in SAM availability for AHL biosynthesis are likely to impact on AQ biosynthesis. In addition, QS will be influenced by the turnover of QSSMs. AHLs, for example, are readily inactivated by pH-dependent lactonolysis and will form the corresponding ring-opened homoserine lactone [27]. For example, *N*-(3-oxododecanoyl)-l-homoserine (3OC12-HS) will be formed from 3OC12-HSL [27] (Fig. 1). 3OC12-HSL can also be inactivated by amide bond cleavage via the endogenous acylase PvdQ [28], whereby it undergoes chemical rearrangment to form the tetramic acid [3-(1-hydrocydecyclidene)−5-(2-hydroxyethyl) pyrrolidine-2, 4-dione; TMA] [28] (Fig. 1). TMA is an iron chelator that inhibits *agr*-dependent QS in *Staphylococcus aureus* and the growth of Gram-positive bacteria [29, 30]. Biosynthesis and turnover of QSSMs will likely impact on metabolism directly via the substrates required for their production and indirectly via metabolic pathways regulated by QS.

As a metabolically versatile, highly adaptable opportunistic pathogen predominantly found in environments associated with human activity [31], *P. aeruginosa* is likely to grow at different rates on diverse but limiting nutrient sources that feed into a variety of metabolic pathways. Growth environment will therefore have a profound impact on AHL and AQ biosynthesis, profile, turnover and hence QS-dependent gene regulation and the QS hierarchy. QSSM production and profile have primarily been investigated using batch cultures in rich laboratory media where they are synthesized at the onset of stationary phase [7, 8, 27]. Apparent population density-dependent phenomena may be related to nutrient limitation or changes in other physico-chemical environmental conditions. Duan *et al*., for example, examined the magnitude and timing of *las* and *rhl* expression in different growth media and observed considerable variation but no direct correlation with population density [8].

In contrast to batch culture, continuous culture enables the growth rate to be varied while maintaining the bacterial cells in a constant growth environment or a specific growth rate to be sustained while varying population density via factors such as nutrient availability, oxygen and temperature. In continuous culture, bacterial growth rate is varied by changing the dilution rate (*D*). This is because growth rate (μ) is determined by the flow rate and consequently by *D*. At steady state, μ is equal to *D*. Limiting the concentration of a specific nutrient (e.g. C, N, P, S, Fe and Mg) at a particular *D* allows population density to be varied by changing the limiting nutrient concentration. Early studies of *P. aeruginosa* exoprotease, elastase and rhamnolipid production (which we now know are QS controlled [4]) in continuous culture highlighted the effect of growth rate and limiting specific nutrients [32–34]. With respect to the Las and Rhl QS systems, Mellbye and Schuster [35] used both batch and continuous culture to investigate nutrient limitation as a trigger for the expression of *las* and *rhl* controlled extracellular enzymes and metabolites. They concluded that expression was unrelated to cell density and was induced only when the limiting nutrient was not a building block of the product. Slow growth and limitation of specific nutrients were noted as key conditions for inducing certain QS-regulated genes. In the present study, we explored how, in a chemically defined minimal medium, growth rate, population density, C source, nutrient limitation (C, N, P, S, Fe, Mg), temperature and anaerobiosis modulated the production and profile of AHLs, AQs and 3OC12-HSL turnover products in *P. aeruginosa *. These compounds were quantified using liquid chromatography/tandem mass spectrometry (LC-MS/MS). We also quantified the impact of inactivation of QS by mutation of individual or multiple AHL and AQ synthases on QSSM profiles, on key QSSM substrates from the activated methyl cycle and aromatic amino acid biosynthesis and on cellular energetics by measuring ATP levels.

## Methods

### Bacterial strains and culture conditions

*P. aeruginosa* PAO1 and isogenic *lasI*, *rhlI*, *pqsA* single, *lasI rhlI* double and *lasI rhlI pqsA* triple mutants have been described previously [14] and were routinely grown in LB or agar at 37 °C. To investigate the impact of population density, growth rate and nutrient limitation, bacterial strains were grown at 37 °C or 25 °C in continuous culture in a 2 l glass fermenter (Sartorius Stedim Biotech). The basal chemically defined medium (CDM) used was previously described by Ombaka *et al*. [32] and contained 20 mM d-glucose, 3 mM KCl, 3 mM NaCl, 12 mM (NH_4_)_2_SO_4_, 3.2 mM MgSO_4_·7H_2_O, 0.02 mM FeSO_4_·7H_2_O, 1.2 mM K_2_HPO_4_ and 50 mM 3-(*N*-morpholino) propanesulfonic acid (MOPS). Foaming was controlled by addition of 0.05 g l^−1^ antifoam 201. The OD_600_ was continuously monitored using a turbidity probe and an automated pH control system maintained pH at 7.2±0.05. The dissolved oxygen concentration was monitored using an O_2_ electrode and maintained at 15±1 % of air saturation. The fermenter was fed with growth medium at dilution rates (*D*) of 0.05, 0.15 or 0.31 h^−1^, which resulted in doubling times of 13.3, 4.62 or 2.23 h, respectively. Population density was modified as required by limiting specific nutrients including glucose (6 mM), nitrogen (0.9 mM), phosphate (0.1 mM), iron (no addition), magnesium (0.016 mM). Sulfur-limited CDM was achieved by replacing the sulphate-containing salts as described by Ombaka *et al*. [32] and contained 0.0225 mM (NH_4_)_2_SO_4_, 0.16 mM MgCl_2_·6 H_2_O, 0.27 mM FeCl_3_·4 H_2_O, 20 mM glucose, 3 mM KCl, 3 mM NaCl, 18.52 mM (NH_4_) 2HPO_4_ and 0.48 mM NH_4_H_2_PO_4_. To evaluate the influence of carbon source on QSSM production, glucose was replaced by glycerol or succinate at concentrations that provided the equivalent number of carbon atoms per molecule as follows: glucose (20 mM), glycerol (40 mM) and succinate (30 mM). For some experiments succinate was also supplied at 20, 45 or 90 mM. For anaerobic growth, *P. aeruginosa* was grown in CDM supplemented with 50 mM KNO_3_ under a continuous flow of nitrogen gas at 2.5 l min^−1^. Samples were collected at steady state after replacement of three culture volumes of fresh medium.

### AQ and AHL extraction and LC-MS/MS analysis

The extraction and LC-MS/MS analysis of AQs and AHLs in cell-free culture supernatants were performed as described by Ortori *et al*. [14]. These experiments were conducted on a 4000 QTRAP hybrid triple-quadrupole linear ion trap mass spectrometer (Applied Biosystems) equipped with a TurboIon source used in positive ion electrospray mode. Analyst (version 1.4.1) was used for data acquisition and processing. All QSSM standards were synthesized in-house as described before [14].

### Intracellular metabolite analysis

AMC metabolites were extracted from bacterial cell pellets and analysed as described by Halliday *et al*. [36]. Briefly, bacterial cells were resuspended in 80 % cold (v/v) methanol containing a mixture of *S*-adenosylcysteine and ^13^CD_3_-methionine as internal standards and lysed by successive cycles of freeze–thaw. Intracellular metabolites were separated from cell debris by centrifugation, and dried extracts derivatized with iso-butyl chloroformate. Extracted derivatized metabolites were then analysed by LC-MS/MS. The AQ-related intracellular metabolites, anthranilate, tryptophan and tyrosine were extracted with chloroform from cells after freeze–thaw lysis. Both hydrophobic and hydrophilic phase components were collected after phase separation, resuspended in isopropanol or water and subjected to LC-MS analysis on a modular Accela HPLC system with an Exactive mass electrospray detector (Thermo Scientific) operated in +/− switching mode. Water and acetonitrile both modified with 0.1 % formic acid were used as the mobile phases with a Gemini C18 (Phenomenex) column. Six technical samples were subjected to analysis for each of three biological replicates. Data were processed using Progenesis CoMet software.

### Quantification of ATP

For ATP quantification *P. aeruginosa* cells were harvested during steady state growth and assayed using a BacTiter-Glo Microbial Cell Viability Assay (Promega UK, Southampton, UK).

### Principal component analysis (PCA)

The large dataset derived from QSSM profiling was analysed by PCA, a clustering method that reduces the dimensionality of the raw data while preserving most of the variances in a two-dimensional map, using Simca-P software (Umetrics, Sweden). The values corresponding to three biological and six technical replicate samples harvested from continuous culture process were used for the PCA.

### Statistical analysis

All QSSM and metabolite analysis experiments were performed with three biological and six technical replicates. Results are presented as means±sem. Statistical calculations and tests were performed using one-way analysis of variance (ANOVA) where appropriate.

## Results and discussion

### Cell population density and growth rate independently modulate QSSM production

To investigate the respective impact of growth rate and cell population density on QSSM accumulation in the extracellular environment, *P. aeruginosa* was grown to steady state in continuous culture in a simple chemically defined minimal medium (CDM) at two different growth rates of µ=0.05 and µ=0.31 h^−1^, i.e. with doubling times of 13.3 (slow) and 2.23 h (fast), respectively. Under these conditions, cell population density was sustained at an OD_600_ of ~1.1–1.3. To maintain *P. aeruginosa* at the same growth rate but at a lower population density, the carbon source concentration (glucose, 20 mM) was reduced to 6 mM. At this limiting glucose concentration the OD_600_ was maintained between 0.29 and 0.38. These parameters were selected to enable us to uncouple growth rate from population density to evaluate their relative impact upon QSSM production. AHLs and AQs were measured in the cell-free culture supernatants using LC-MS/MS. The complete dataset is summarized in Table S1 (available in the online version of this article).

**Fig. 2.**
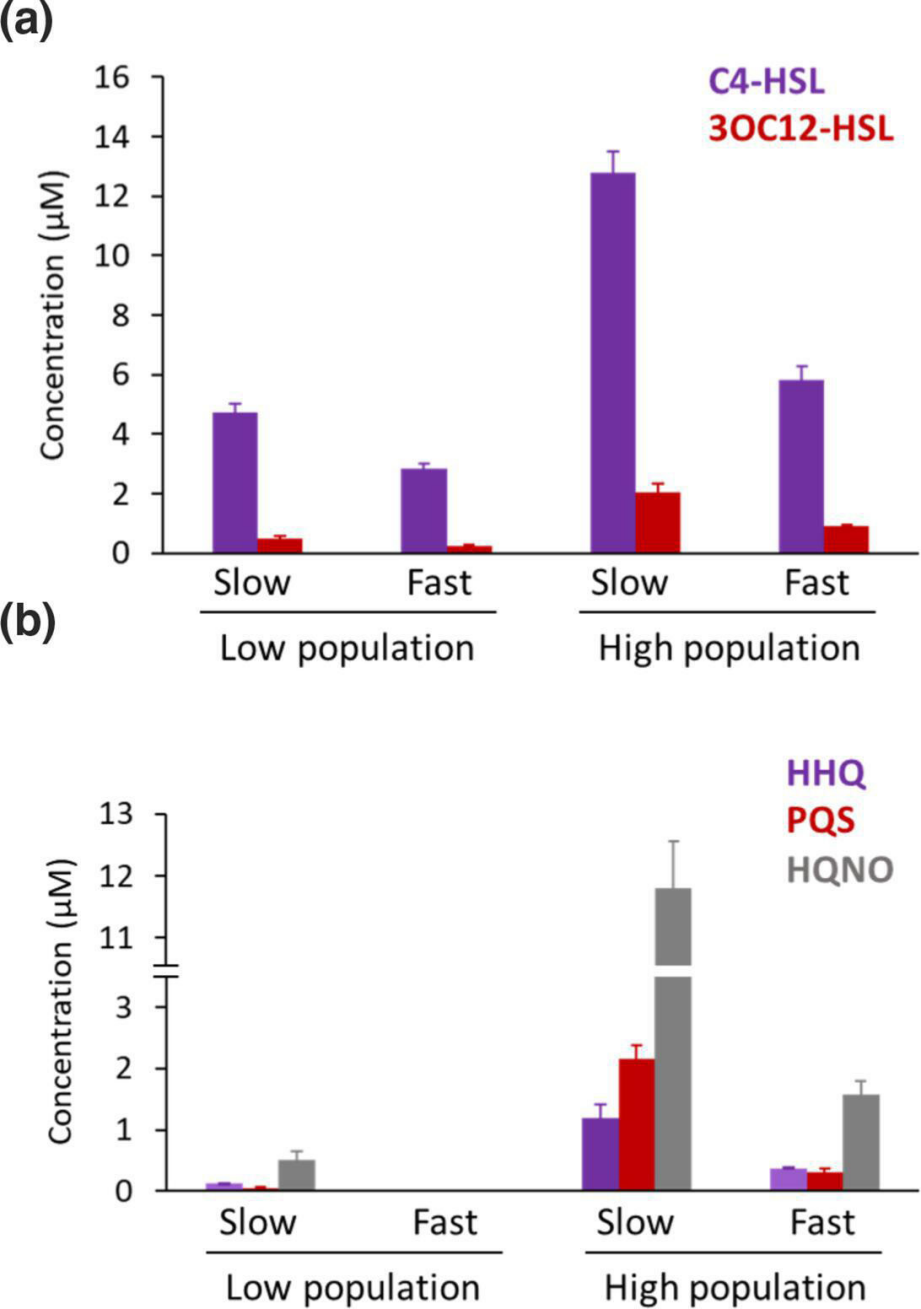
Population density and growth rate both influence QSSM biosynthesis. *P. aeruginosa* was grown to steady state at a slow (µ = 0.05 h^-1^) or fast (µ = 0.31 h^-1^) growth rate in CDM where population density was maintained at a OD_600_ ~0.3 (low) or OD_600_ ~1.1 (high). QSSMs were solvent-extracted and quantified using LC-MS/MS. (a) C4-HSL and, 3OC12-HSL and (b) HHQ, PQS and HQNO**.** SDs are based on the mean of six samples taken at steady state.

Previously, Ortori *et al.* [14] reported that when *P. aeruginosa* was grown to stationary phase (24 h) in a 2 l glass fermenter in batch culture and complete CDM, C4-HSL and 3OC12-HSL concentrations reached 5.6±0.4 and 1.4±0.12 µM, respectively. In contrast, in continuous culture, C4-HSL production ranged from 2.8±0.17 to 12.8±0.7 µM, with the highest concentration being produced at slow growth rate and high population density (Fig. 2a and Table 1). C4-HSL levels were considerably greater (up to 6.3-fold) than those of 3OC12-HSL under all conditions tested (Table 1). This may, in part, reflect the greater solubility of the short-chain C4-HSL [27] and the higher EC_50_ for RhlR compared with LasR [11–13]. Fig. 2a shows that for C4-HSL and 3OC12-HSL, higher levels of both AHLs were produced at high compared with low population density. Greater concentrations of AHLs were produced at the slow growth rate for both population densities, indicating that both cell population density and growth rate independently affect AHL production.

**Table 1.** Quantification of the major QSSMs in cell-free culture supernatants of *P. aeruginosa* PAO1 Bacteria were grown aerobically or anaerobically in continuous culture at 37 °C (or 25 °C) at µ=0.05 (slow) or µ=0.31h^−1^ (fast) in complete or nutrient-limited CDM.

Growth condition	Growth rate (μ)	OD_600_	C4-HSL (μm)	3OC12-HSL (μm)	HHQ (μm)	PQS (μm)	HQNO (μm)
Complete	Slow	1.13	12.8	2.04	1.2	2.16	11.3
	Fast	1.36	5.8	0.93	0.38	0.3	1.58
Carbon	Slow	0.38	4.7	0.49	0.12	0.05	0.5
	Fast	0.29	2.8	0.23	<0.01	<0.01	0.09
Nitrogen	Slow	0.21	4.2	0.25	0.12	<0.01	<0.01
	Fast	0.20	2.4	0.17	0.04	<0.01	<0.01
Phosphate	Slow	0.66	6.7	0.51	3.52	6.00	100.7
	Fast	0.53	5.6	0.48	1.8	3.2	38.8
Sulfur	Slow	0.3	1.26	0.03	2.9	3.8	122.0
	Fast	0171	0.65	0.08	0.3	0.2	4.8
Iron	Slow	1.29	1.6	0.024	0.57	1.43	7.4
	Fast	1.02	0.8	0.04	0.59	1.01	1.81
Magnesium	Slow	1.39	4.8	0.21	0.43	0.52	0.52
	Fast	1.54	1.5	0.35	0.34	0.19	0.11
Anaerobic	Slow	0.18	2.12	0.24	0.13	0.26	0.45
	Fast	0.15	0.2	<0.01	0.04	<0.01	0.10
25 °C	Slow	1.02	9.33	0.42	0.03	0.06	0.77
	Fast	0.58	6.04	0.45	0.02	0.04	0.54

Our data contrast with those of Mellbye and Schuster [35] who reported that 3OC12-HSL concentrations were higher at faster growth rates (µ=0.5 h^−1^ and 0.33 h^−1^) than C4-HSL but lower at slower growth rates (µ=0.25 h^−1^ and 0.13 h^−1^). However, the data in [35] were obtained under phosphate limiting conditions with glutamate as the carbon source and these changes may account for the differences observed. In addition the presence of 3OC12-HSL antagonizes the responses of *Escherichia coli* based AHL bioassays for C4-HSL [37] and this may also have influenced the AHL data reported by [35].

With respect to the AQs, the impact of both parameters on the production of HHQ, PQS (Fig. 2b and Table 1) was even more marked; HHQ and PQS increased by 10- and 20-fold during fast and slow growth rates respectively at the high compared with the low population density (Fig. 2b). It is noteworthy that HQNO which is not involved in QS is produced at much higher concentrations than the QS signals PQS and HHQ (Fig. 2b). This probably reflects their differential functions; the AQ *N*-oxides can act as antibiotics for Gram-positive bacteria, cytochrome inhibitors for both mammalian and microbial cells and drivers of autolysis in *P. aeruginosa* [23].

**Fig. 3.**
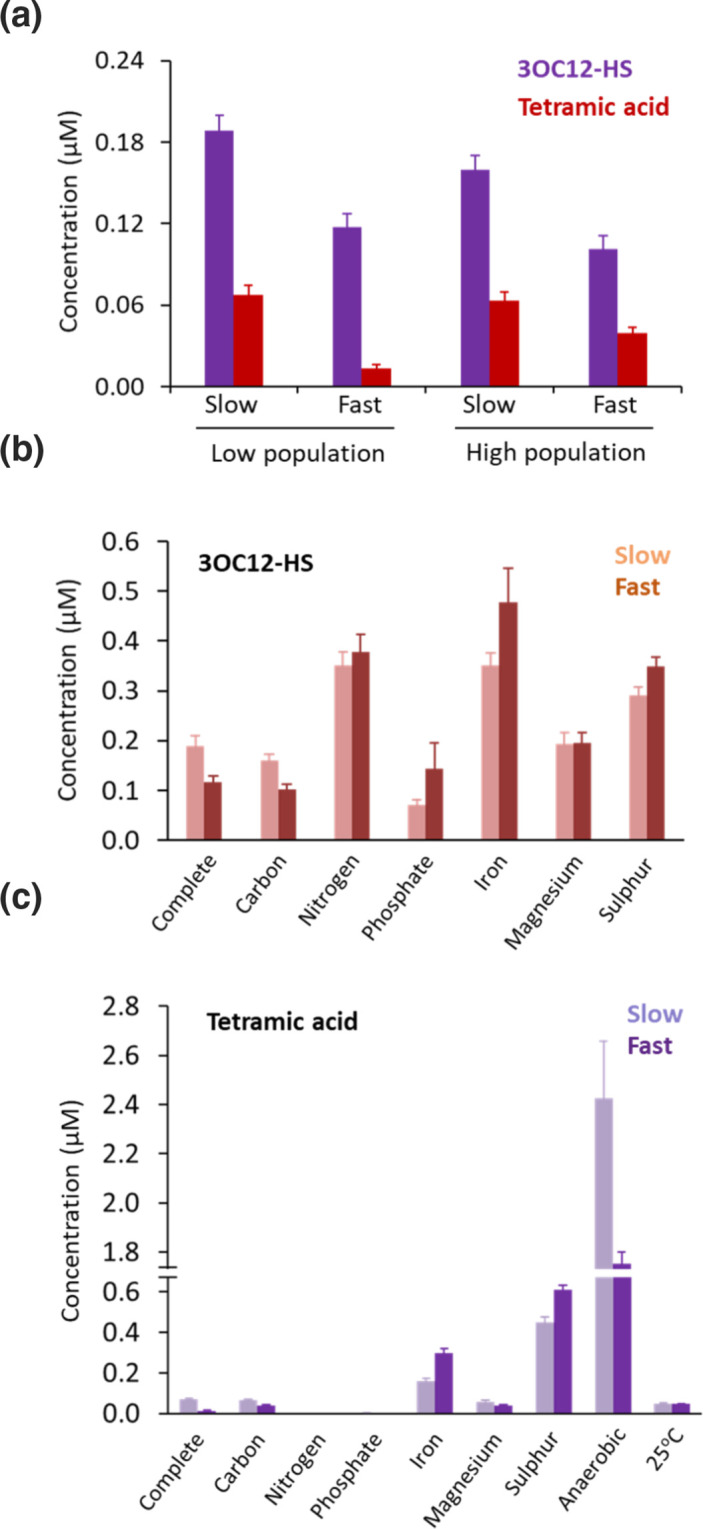
Inactivation of 3OC12-HSL to form the corresponding open ring compound and tetramic acid during continuous culture. *P. aeruginosa* was grown to steady state at a slow (µ=0.05 h^−1^) or a fast (µ=0.31 h^−1^) growth rate where population density was maintained at a OD_600_ ~0.3 (low) or OD_600_ ~1.1 (high). The ring open form of 3OC12-HSL and tetramic acid were solvent extracted and quantified using LC-MS/MS. SDs are based on the mean of six samples taken at steady state.

3OC12-HSL is at the top of QS hierarchy in *P. aeruginosa* and can be inactivated to form 3OC12-HS and TMA via chemical rearrangement [27, 29, 30]. LC-MS/MS analysis of spent culture supernatants revealed the presence of both 3OC12-HS and TMA at low concentrations that were 9 and 3.4 % of the 3OC12-HSL concentration at the slow growth rate and high population density respectively (Fig. 3 and Table 2). These low levels are likely a consequence of the well buffered growth conditions maintained at pH 7.2 since formation of the 3OC12-HSL turnover products is favoured by more alkaline pHs [27, 29].

**Table 2.** Quantification of the 3OC12-HSL turnover products 3OC12-HS and TMA in cell free culture supernatants of *P. aeruginosa* PAO1 Bacteria were grown aerobically or anaerobically in continuous culture at 37 °C (or 25 °C) at µ=0.05 h^−1^ or µ=0.31h^1^ in complete or nutrient limited CDM.

Growth condition	Growth rate (μ)	OD_600_	3OC12-HSL (μM)	3OC12-HS (μM)	TMA (μM)
Complete	Slow	1.13	2.04	0.19	0.07
	Fast	1.36	0.93	0.12	0.01
Carbon	Slow	0.38	0.49	0.16	0.06
	Fast	0.29	0.23	0.10	0.04
Nitrogen	Slow	0.21	0.25	0.35	<0.01
	Fast	0.20	0.17	0.38	<0.01
Phosphate	Slow	0.66	0.51	0.07	<0.01
	Fast	0.53	0.48	0.14	<0.01
Sulfur	Slow	0.30	0.03	0.29	0.45
	Fast	0.17	0.08	0.35	0.61
Iron	Slow	1.29	0.02	0.35	0.16
	Fast	1.02	0.04	0.48	0.30
Magnesium	Slow	1.39	0.21	0.19	0.06
	Fast	1.54	0.35	0.19	0.04
Anaerobic	Slow	0.18	0.24	nd	2.4
	Fast	0.15	<0.01	nd	1.75
Temperature	Slow	1.02	0.42	nd	0.05
	Fast	0.582	0.45	nd	0.05

ND, not determined.

### Carbon source influences QSSM production and profile

**Fig. 4.**
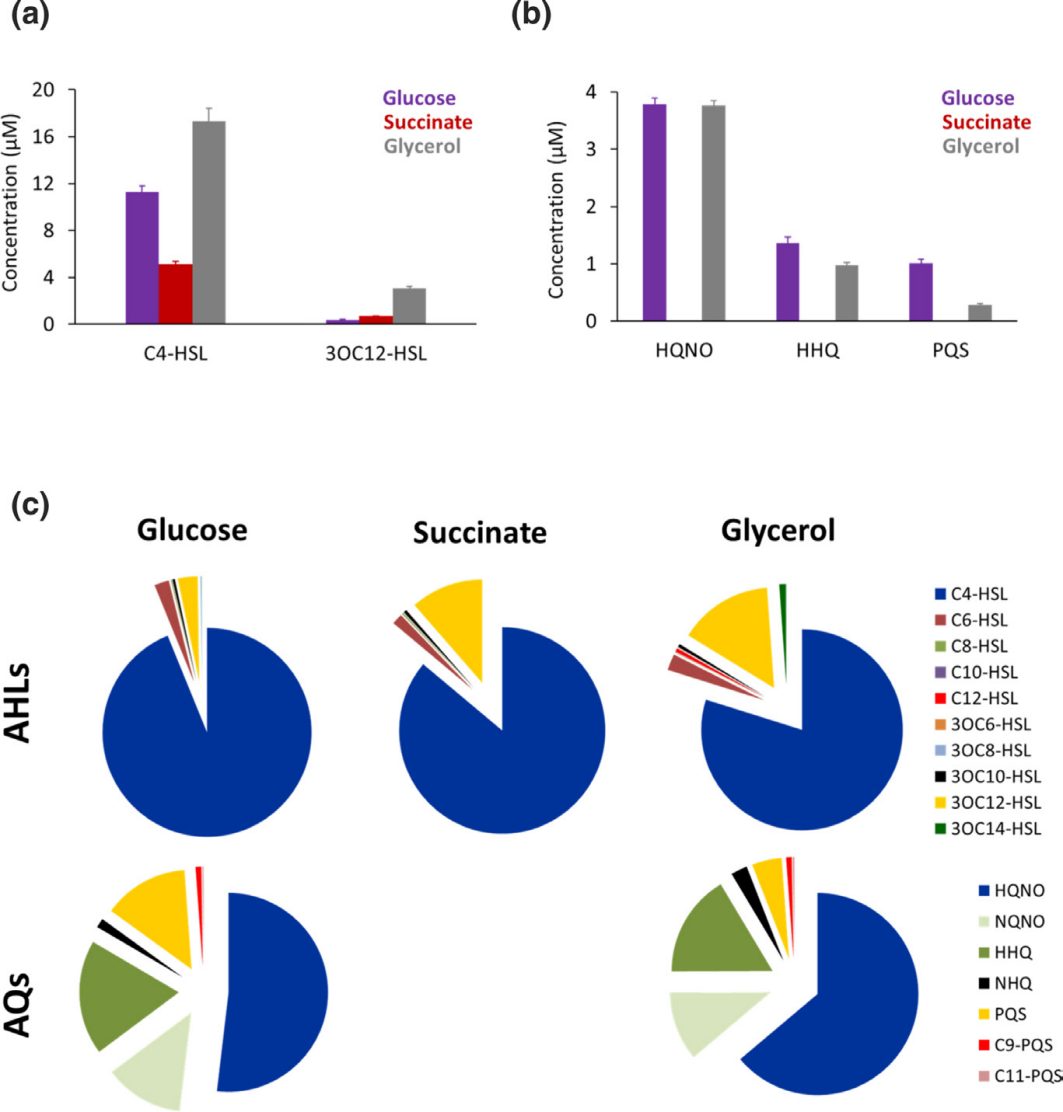
Quantitative profiling of the QSSMs produced by *P. aeruginosa* grown in continuous culture containing alternative carbon sources. *P. aeruginosa* was grown to steady state at µ=0.15 h^−1^ in CDM containing glucose (20 mM), glycerol (40 mM) or succinate (30 mM) at the equivalent amounts of carbon. QSSMS were extracted and profiled by LC-MS/MS analysis. (**a**) AHLs (**b**) AQs and (**c**) AHL and AQ profiles. sds are the means values of six samples taken at steady state.

Carbon source is known to induce distinct metabolic regimes in pseudomonads [38]. Glycerol, for example, stimulates rhamnolipid production [39], the synthesis of which is dependent on both the *rhl* and *pqs* systems. We therefore determined whether QSSM concentrations and profiles in continuous culture varied according to the carbon source when *P. aeruginosa* was cultured in CDM, under aerobic conditions at pH (7.2) with either glucose (20 mM; 6C), glycerol (40 mM; 3C), or succinate (30 mM; 4C). For each carbon source, the concentration was selected to provide an equivalent number of carbon atoms, since each source feeds into QSSM biosynthesis via a different metabolic entry pathway: glucose (Entner–Doudoroff); glycerol (phosphorylated and converted to dihydroxy acetone phosphate prior to feeding into central carbon metabolism [39]) and succinate (TCA cycle). With an intermediate dilution rate of µ=0.15 h^−1^, the cell population densities (OD_600_) achieved at steady state were 1.25±0.03 (glucose), 1.6±0.07 (glycerol) and 0.9±0.01 (succinate). Fig. 4 shows how the QSSM profiles were influenced by the carbon source supplied. On glucose, the concentrations of C4-HSL and 3-oxo-C12-HSL were 11.26±0.5 and 0.36±0.03 µM, respectively, while the AQs, HQNO, HHQ and PQS were 3.8±0.10, 1.36±0.11 and 1.0±0.06 µM. Most notably, growth on glycerol generated the highest AHL levels, 17.56 µM C4-HSL and 3.05 µM 3OC12-HSL (Fig. 4a). In addition, whereas the HQNO and HHQ concentrations produced were similar when *P. aeruginosa* was grown on either glycerol or glucose, PQS production on glycerol was substantially reduced by ~3.6-fold (Fig. 4b).

**Fig. 5.**
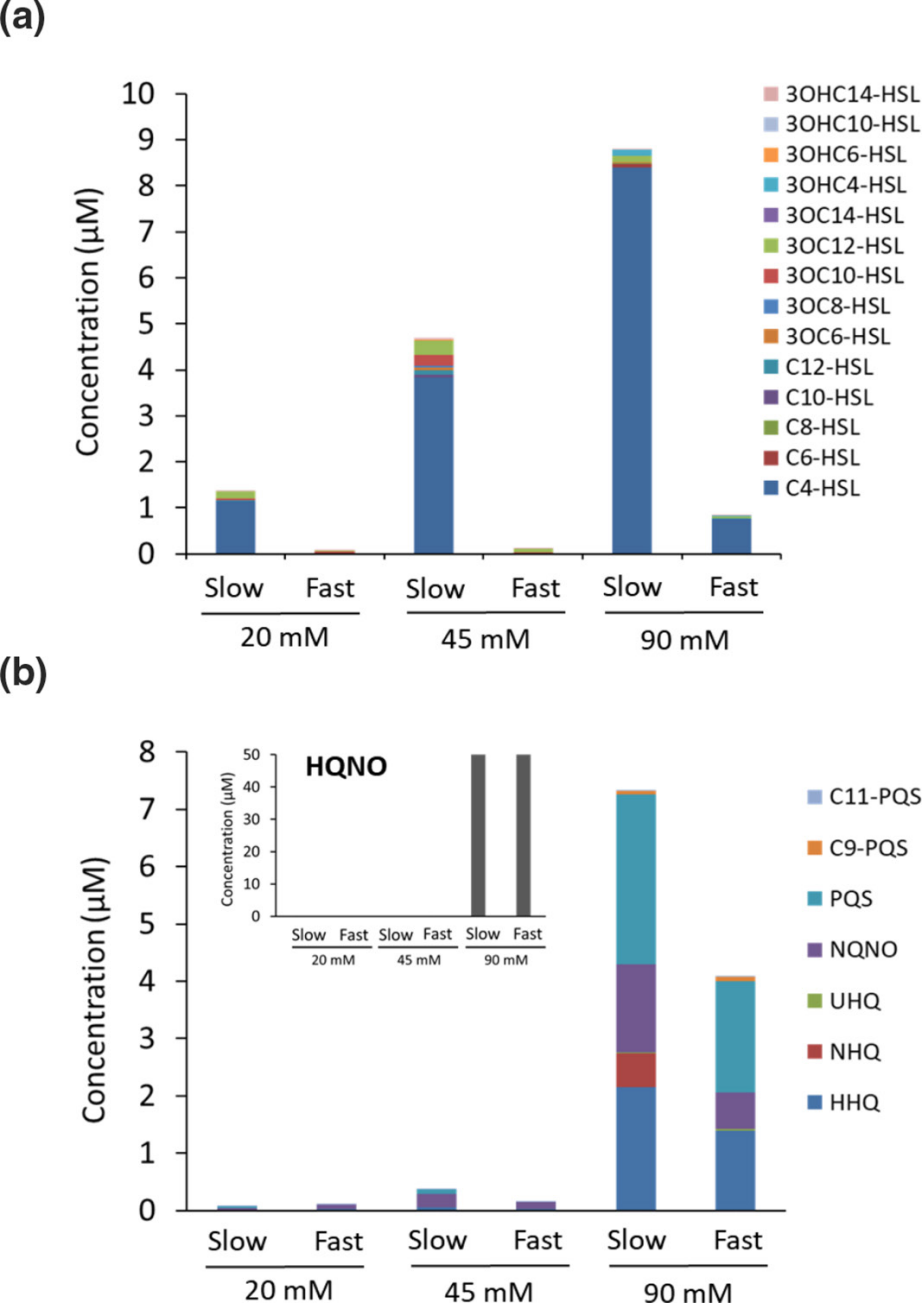
*
P. aeruginosa* QSSM profile during continuous culture at slow (µ=0.05 h^−1^) or fast (µ=0.31 h^−1^) growth rate in CDM containing succinate at 20, 45 or 90 mM. QSSMs were extracted and profiled using LC-MS/MS. (**a**) AHLs and (**b**) AQs. HQNO data are presented in the inset because of the high concentration attained. sds are based on the means values of six samples taken at steady state.

In contrast, growth on succinate at equivalent concentrations to glucose and glycerol resulted in a ~50 % reduction in AHL levels and a complete loss of AQ production (Fig. 4b, c). Since succinate supplied at 30 mM resulted in a reduced steady state cell population density (OD_600_ 0.9), ~50 % reduction in AHLs and in the loss of AQ production (Fig. 4), we explored the impact of succinate supplied at 20, 45 and 90 mM in the context of dilution rate and population density (Fig. 5). At the fast growth rate (µ=0.31 h^−1^), total AHL production at 20 and 45 mM succinate was almost undetectable but reached ~1.0±0.18 µM in 90 mM succinate (Fig. 5a). At the slow dilution rate (µ 0.05 h^−1^), AHL production reached similar levels at the lowest concentration of succinate (20 mM) provided, but increased by ~4- and ~8-fold, respectively, at the two higher population densities where the OD_600_ reached for the different succinate concentrations was 1.1 for 45 mM and 1.4 for 90 mM. The relative concentrations of C4-HSL and 3OC12-HSL at the different succinate concentrations were 3.83±0.07 and 0.31±0.02 µM at 45 mM succinate, and 8.40±0.42 and 0.14±0.01 µM at 90 mM succinate, respectively.

While increasing the succinate concentration to 45 mM had little impact on AQ levels at either the slow or fast dilution rate, AQ accumulation was restored at 90 mM succinate for both growth rates, with a ~2-fold higher concentration produced at the slow growth rate (Fig. 5b). Thus three times more carbon was required to produce approximately the same AQ concentration when succinate was supplied instead of glucose.

### QSSMs, growth rate, nutrient limitation and temperature

To explore the influence of nutrient limitation and growth rate, *P. aeruginosa* was grown in continuous culture at µ=0.05 or 0.31 h^−1^ at 37 °C in complete CDM or CDM in which C, N, P, S, Mg or Fe were limiting. The impact of temperature was also investigated by comparing QSSM production in complete CDM at 37 and at 25 °C for both growth rates. Tables 1 and S1 summarize the data obtained. PCA revealed that around 30 % of the variation observed across the dataset was due to the growth rate, whereas a further 26 % variation was due to nutrient limitation (Fig. S1).

In general, limitation of a specific nutrient or growth at 25 °C resulted in variable reductions in QSSM levels when compared with growth in complete CDM (Tables 1 and S1). QSSM production was mostly substantially lower at the higher growth rate. No switches in the ratio of C4-HSL to 3-OC12-HSL were observed for any of growth conditions tested, in contrast to what was reported in [35]. Regulatory links between QS and iron are well established in the literature, with both *rhlRI* and *lasRI* expression and C4-HSL production reportedly enhanced by iron depletion [40]. AQ biosynthesis is regulated in low-iron conditions by two small RNAs, PrrF1 and PrrF2, although PQS levels in the wild-type PAO1 were reduced in iron-depleted TSB medium [41]. In our continuous culture experiments where Fe-limited and Mg-limited cultures at slow or fast growth rates reached similar population densities to the complete CDM, at µ=0.05 h^−1^, under Fe limitation, 3OC12-HSL was reduced by ~100-fold, C4-HSL by 8-fold and the AQs by ~2-fold. Table 1 shows that a fast growth rate exerted a much greater effect than iron limitation on HQNO levels. When grown to stationary phase in a low-Mg minimal medium containing glycerol and casamino acids [41], PAO1 reportedly showed increased expression of *lasI* and the *pqs* operon, together with a ~5-fold increase in PQS (as determined by thin layer chromatography) [42]. However in Mg-limited CDM at the slow growth we observed a reduction of ~10-fold in 3OC12-HSL, ~2.7-fold for C4-HSL and ~4-fold for PQS, 2.8-fold for HHQ and >20-fold for HQNO (Tables 1 and S1). The changes observed for Mg limitation are likely to be due to the differences in the growth media and culture conditions used.

In contrast to Fe and Mg limitation, S and P limitation notably resulted in greatly enhanced production of the AQs, especially HQNO (from 11.3 µM in complete CDM to 122 and 100.7 µM, respectively, at µ=0.05 h^−1^), whereas both C4-HSL and 3-OC12-HSL production were reduced (Table 1). A *P. aeruginosa* PAO1 sulfur starvation regulon has been characterized but does not contain genes known to be associated with QS [43]. Under phosphate deficiency, where the *P. aeruginosa* QS circuitry is controlled by the PhoRB two-component system, *rhlR* and *pqsR* expression and PQS production are reportedly enhanced, consistent with the increased AQ production observed [44, 45]. P deficiency is relevant to *P. aeruginosa* infections, where host phosphate levels are low and reportedly enhance activation of a hypervirulent phenotype [46].

Both *rhlR* and *lasI* were previously found to be post-transcriptionally thermoregulated via two RNA thermometers [47]. In contrast, the data presented here show that reducing the incubation temperature to 25 °C at either growth rate had relatively little effect on C4-HSL and reduced 3OC12-HSL levels (4.8-fold at µ=0.05 h^−1^; OD_600_ 1.02), but virtually abolished AQ production [40-fold reduction for HHQ, 36-fold for PQS and 14.7-fold for HQNO; similar for both growth rates (Table 1)].

PCA (Fig. S1) revealed that ~30 % of the variation across the dataset was due to the growth rate, whereas a further 26 % variation was due to nutrient limitation. Concentrations of the minor AHLs and AQs, produced in continuous culture under the different growth conditions largely mirrored the changes observed for the major QSSMs (Tables 1 and S1). With respect to the 3OC12-HSL turnover products, 3OC12-HS levels were ~2-fold higher under N and Fe limitation than in complete CDM at either growth rate (Fig. 3b and Table 2), whereas TMA was substantially increased in anaerobic growth conditions(Fig. 3c and Table 2); by 36-fold and 134-fold at slow and fast growth rates, respectively. This suggests that although substantial levels of 3OC12-HSL must have been produced under anaerobic conditions, this is turned over to generate TMA.

### QS synthase inactivation modifies QSSM profile – effect of exogenous QSSMs

**Fig. 6.**
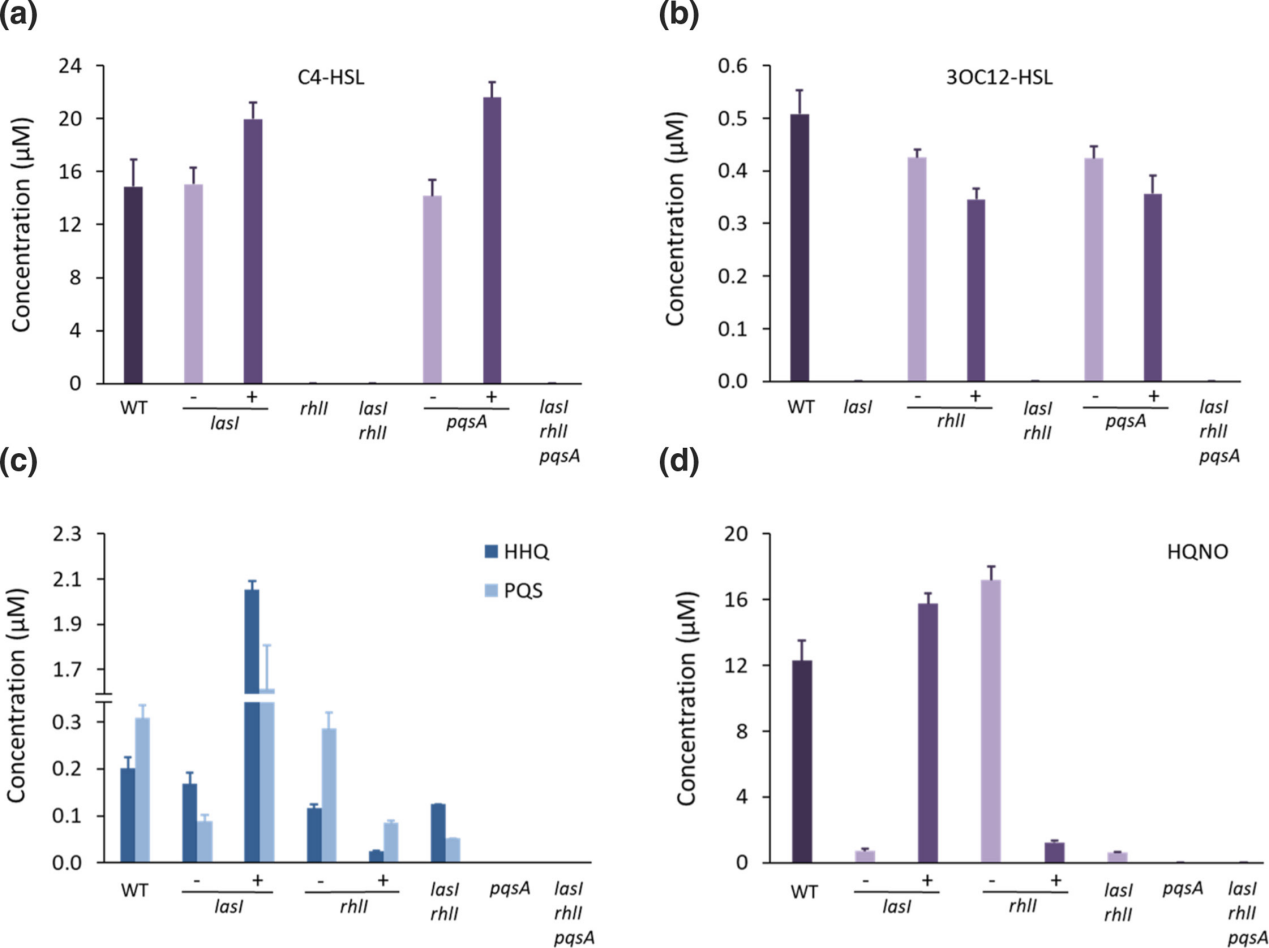
QSSM profiles of *P. aeruginosa* wild-type compared with single *lasI*, *rhlI* and *pqsA* mutants, a *lasI rhlI* double mutant and a *lasI rhlI pqsA* triple mutant grown in continuous culture at µ=0.15 h^−1^. (**a**) C4-HSL (**b**) 3OC12-HSL), (**c**) HHQ, PQS and (**d**) HQNO. Where indicated, exogenous 3OC12-HSL(1 µM), C4-HSL (10 µM) or PQS (5 µM) was added. AHLs and AQs were quantified using LC-MS/MS. Values represent the means of three biological replicates and six technical replicates. +, exogenous addition of the cognate QSSM.

To investigate the influence of continuous culture on QSSM production and the impact of exogenous provision of the cognate QSSM, we grew *P. aeruginosa* strains with mutations in *lasI, rhlI*, *pqsA,* a double *lasI rhlI* and a triple *lasI rhlI pqsA* mutant to physiological steady state in complete CDM, high population density and an intermediate growth rate of µ=0.15 h^−1^ (doubling time, 4.62 h). Mutants in *lasI*, *rhlI*, *pqsA*, the double *lasI rhlI* and the triple *lasI rhlI pqsA* mutants grew to an OD_600_ of ~1.1, ~1.2, ~1.6, ~1.3 and ~1.6, respectively when compared with the wild-type (OD_600_ of ~1.1), indicating that loss of QS and particularly the PQS system resulted in an increase in population density at this dilution rate in CDM. LC-MS/MS confirmed that each of the mutants were unable to produce the cognate QSSM(s) with respect to the synthase gene(s) mutated. For example, as expected, no AHLs were produced by the *lasIrhlI* mutant; no AQs by the *pqsA* mutant; and no AHLS or AQs by the *lasI rhlI pqsA* mutant. Disruption of *lasI* abolished 3OC12-HSL, had no effect on C4-HSL production but reduced PQS by 3-fold and HQNO by 12-fold (Fig. 6). Exogenous addition of 3OC12-HSL to the *lasI* mutant culture substantially increased the levels of HHQ, PQS and HQNO by over 10-fold compared with the wild-type (Fig. 6). Deletion of *rhlI* abolished C4-HSL with little impact on 3OC12-HSL or the AQs. However, AQ production was reduced by ~50 % in the *lasI rhlI* double mutant (Fig. 6), whereas exogenous addition of C4-HSL drastically reduced the synthesis of AQs, especially HQNO (~17-fold) (Fig. 6). These data are consistent with the activation and repression of the *pqs* system by the *las* and *rhl* systems, respectively, and their regulatory interdependence [4, 5].

### QSSM synthase inactivation correlates with reduced intracellular AMC intermediates, anthranilate and aromatic amino acids

**Fig. 7.**
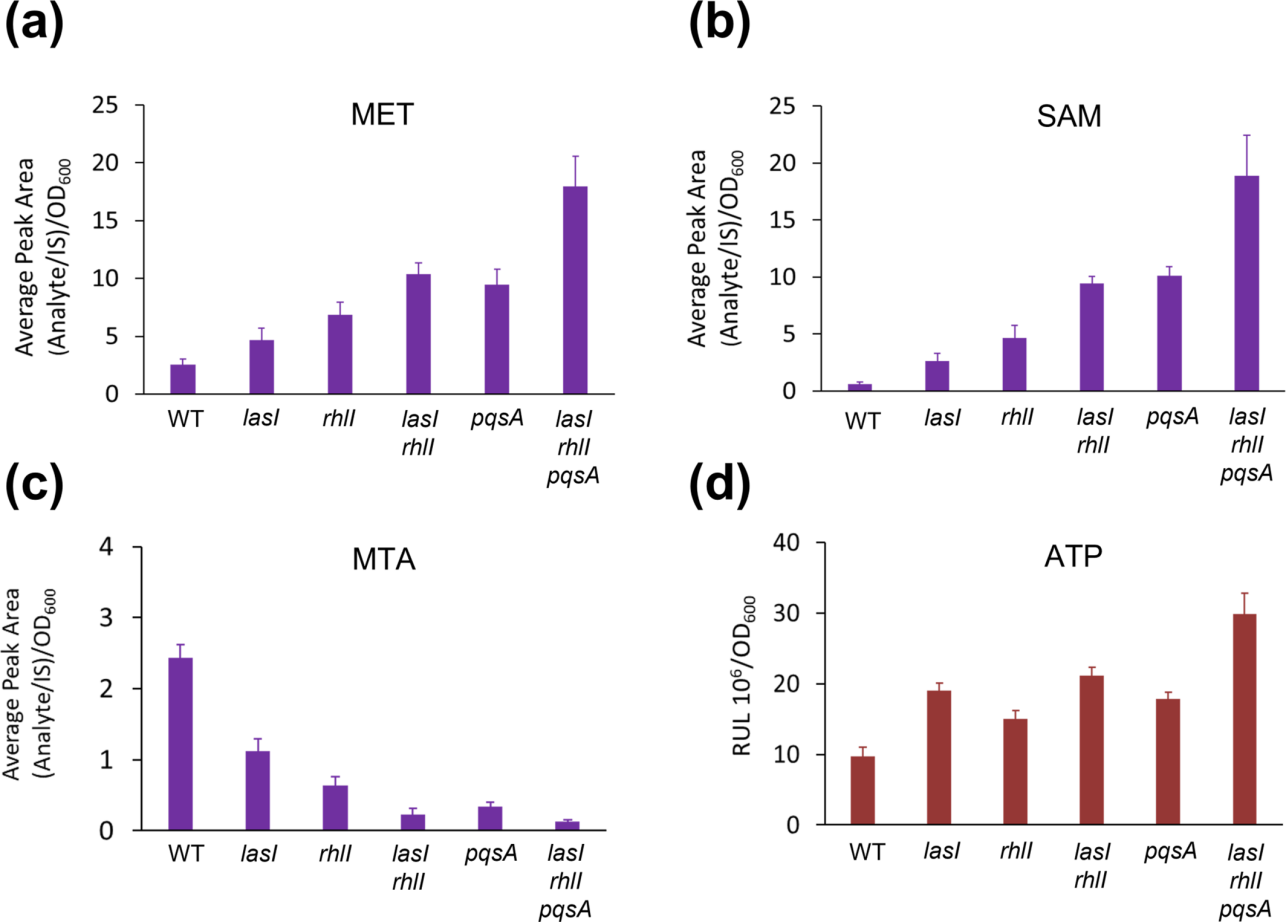
QS influences AMC pathway metabolite levels and cellular ATP status of *P. aeruginosa* wild-type compared with single *lasI*, *rhlI* and *pqsA* mutants, a *lasI rhlI* double mutant and a *lasI rhlI pqsA* triple mutant grown in continuous culture at µ=0.15 h^−1^. (**a**) methionine (MET), (**b**) *S*-adenosyl-methionine (SAM), (**c**) 5’-methylthioadenosine (MTA) and (**d**) ATP. Intracellular AMC metabolite levels were quantified by derivatization followed by LC-MS/MS. Values represent the mean ratio between the area of the peak of the AMC metabolite and of the internal standards over cell density (OD_600_) for six samples taken at steady state. ATP was quantified using a bioluminescence detection assay.

When *rhl* or *lasI* are expressed in a heterologous host (* E. coli *) and cultured in a minimal medium, they impose a fitness cost by acting as a drain on AMC pathway metabolites required for AHL biosynthesis [10]. Since AHL biosynthesis depends on the AMC pathway and S-containing amino acids, the reduction in AHL production by *P. aeruginosa* noted during S limitation in continuous culture is consistent with the recombinant *E. coli* data. To determine how mutation of *lasI*, *rhlI* and *pqsA* impacts on key AMC pathway metabolites in their homologous genetic background, intracellular levels of methionine, SAM, and 5′-methythioadenosine (MTA) were quantified in *P. aeruginosa* using chemical derivatization coupled with LC MS [36]. For these experiments, the *P. aeruginosa* wild-type and synthase mutants were grown in the complete CDM to steady state at µ=0.15 h^−1^. In *lasI*, *rhlI* and *lasI rhl* mutants, methionine and SAM levels increased compared with the wild-type by 1.8- and 4.4-fold for the *lasI* mutant, 2.6- and 7.8-fold for the *rhlI* mutant, and 4- and 15-fold for the *lasI rhlI* mutant, consistent with the reduction in production of one or both AHLs (Fig. 7). These data were clearly reflected in the reduced levels of MTA, the metabolite released following donation of the homoserine lactone moiety by SAM during AHL biosynthesis. Although AQ biosynthesis is not SAM-dependent, increases in methionine and SAM and a decrease in MTA levels were also observed in the *pqsA* mutant. This likely reflects the requirement for SAM in *pqs* regulated pathways, including pyocyanin [48] and azetidine alkaloid [49] biosynthesis. The greatest differences in methionine, SAM and MTA levels were, however, observed for the triple *lasI rhlI pqsA* mutant when compared with the wild-type (Fig. 7).

With respect to the AQs, the levels of three metabolites (tryptophan, tyrosine and anthranilate) associated with AQ biosynthesis were quantified by LC-MS for each of the synthase mutants (Table 3). Although similar levels of these intracellular metabolites were present in the wild-type and *lasI* and *rhlI* mutants*,* substantially higher levels of each were present in the *pqsA* mutant, consistent with their requirement for AQ biosynthesis. Since anthranilate can be directed either via the TCA cycle for energy metabolism or into AQ biosynthesis [41], mutation of *pqsA* is likely to redirect it towards the TCA cycle and hence, the AMC.

**Table 3.** LC-MS quantification of intracellular tyrosine, tryptophan and anthanilate levels in *P. aeruginosa lasI*, *rhlI* and *pqsA* mutants compared with wild-type

*m/*z*	Elution time (min)	ES mode	KEGG ID	Compound name	*P*-value (ANOVA)	*lasI*	*rhlI*	*pqsA*
138.055	2.107	+	C00108	Anthranilate	1.91E-28	1.68	1.53	76.64
182.081	2.075	+	C00082	l-tyrosine	4.74E-20	−1.70	−3.14	30.25
205.097	4.108	+	C00078	l-tryptophan	1.11E-13	1.59	1.25	9.95

*Bacteria were grown to high cell density at µ=0.15 h^−1^ in complete CDM. The mean values are based on three samples taken during the steady state and extracted in parallel.

Since these data reflect not only QSSM production but also metabolites from pathways regulated by QS, we assayed the energetic state of wild-type and mutants by quantifying ATP levels and observed that these rose by approximately 1.5-fold in the *rhlI* mutant, 2-fold in the *lasI* and double *rhlI lasI* mutants, 1.8-fold in the *pqsA* mutant and 3-fold in the triple *lasI rhlI pqsA* mutant (Fig. 7d). These data highlight the significant energy costs associated with sustaining an intact, interdependent multi-signal QS hierarchy in *P. aeruginosa *.

### Conclusions

QS as a population density-dependent phenomenon has predominantly been investigated in nutrient-rich batch cultures where bacteria grow rapidly with short generation times. Such conditions are atypical of natural, nutrient-limited environments, including those encountered *in vivo* during infection. Continuous culture offers the possibility of mimicking natural conditions since it allows bacteria as single species or polymicrobial communities to be grown to physiological steady state at different specific growth rates and population densities by varying physico-chemical parameters. This in turn facilitates investigation of the impact of QS on evolution, population dynamics and interspecies interactions as a function of the prevailing growth environment.

*P. aeruginosa *, for example, employs a sophisticated interdependent multi-signal QS regulatory network that controls the expression of genes involved in virulence, secondary metabolism, motility and biofilm development. It produces a diverse range of major and minor AHLs and AQs in laboratory conditions, many of which have been detected, for example in human body fluids in patients infected with *P. aeruginosa* [50].

Not all of the AHLs and AQs are involved in QS, but some, such as the AQ *N*-oxides, offer a competitive advantage over other organisms occupying the same environmental niche [51]. Using continuous culture to uncouple growth rate and population density, we conclude that specific environmental parameters independently influence the accumulation of AHLs and AQs, such that the highest concentrations of each chemical class were observed at a slow growth rate and high population density at 37 °C. AQ levels were more markedly affected by growth environment than the AHLs. Fast growth rates had a negative impact on QSSM accumulation, irrespective of population density. Principal component analysis indicated that ~30 % of the variation across the dataset was due to the growth rate, whereas a further 26 % of variation was due to nutrient limitation. Nutrient limitation or growth at 25 °C generally reduced the extracellular concentrations of AHLs and AQs, except for P- and S- limitation, which resulted in substantially higher concentrations of AQs, especially the AQ *N*-oxides, despite the lower population densities achieved. Formation of the antibacterial 3OC12-HSL turnover product, TMA, was also dependent on nutrient limitation, though the highest extracellular levels were observed during growth under anaerobic conditions. Despite the interconnected nature of the QS hierarchy in *P. aeruginosa *, there was clear differential accumulation of the AHLs and AQs that was dependent on the prevailing growth conditions. Inactivation of QS by mutation of three key genes required for QSSM synthesis (*lasI*, *rhlI* and *pqsA*) substantially increased the concentrations of selected substrates from the AMC and aromatic amino acid pathways, as well as ATP levels, highlighting the energetic drain that QSSM synthesis and hence QS imposes on *P. aeruginosa *.

Further work using continuous culture will be required to understand whether and how specific growth rates and limiting nutrients relevant, for example, to infection modulate the size of the quorum, the sensitivity of the QS signal molecule response and the differential expression of QS-dependent target genes.

## Supplementary Data

Supplementary material 1
