# Supplementary material for: Growth rate and nutrient limitation as key drivers of extracellular quorum sensing signal molecule accumulation in Pseudomonas aeruginosa
Source: Microbiology (Reading). 2023 Apr 5;169(4):001316. doi: 10.1099/mic.0.001316 (PMC10202320; doi:10.1099/mic.0.001316)
Supplement: Supplementary material 1 [file mic-169-1316-s001.pdf]

Supplementary Materials for

Growth rate and nutrient limitation as key drivers of extracellular quorum sensing signal molecule accumulation in *Pseudomonas aeruginosa*

Jean-Frédéric Dubern*, Nigel Halliday, Miguel Cámara, Klaus Winzer, David A. Barrett, Kim R. Hardie, and Paul Williams*

*Corresponding authors. Mailing address: Biodiscovery Institute, University of Nottingham, University Park, Nottingham NG7 2RD, United Kingdom. Emails: jean.dubern@nottingham.ac.uk and paul.williams@nottingham.ac.uk.

Table S1. Quantification of all AHLs and AQs produced by *P. aeruginosa* during growth in continuous culture at slow (μ=0.05 h^{-1}) and fast (μ=0.31 h^{-1}) growth rates with respect to nutrient limitation, anaerobic condition and temperature.

[a]Quorum Sensing Signalling molecule (µM) produced in culture

Limited condition	Dilution rate (hr^{-1})	OD_{600}	Unsubstituted HSL				3-oxo-HSL						3-hydroxy-HSL	4-quinolone			hydroxy-4-quinolone			4-quinolone-*N*-oxide		Open ring C12	Tetramic acid
			C4	C6	C8	C10	C4	C6	C8	C10	C12	C14	C4	C7	C9	C11	C7	C9	C11	C7	C9		
Complete	0.05	1.13	12.80 ± 0.70	0.29 ± 0.02	0.02 ± 0.00	<0.01	<0.01	<0.01	0.01 ± 0.00	0.12 ± 0.02	2.04 ± 0.32	0.01 ± 0.00	0.46 ± 0.05	1.20 ± 0.21	0.23 ± 0.02	0.01 ± 0.00	2.16 ± 0.21	0.10 ± 0.02	<0.01	11.31 ± 0.75	1.68 ± 0.13	0.19 ± 0.02	0.07 ± 0.01
	0.31	1.36	5.82 ± 0.49	0.12 ± 0.01	0.01 ± 0.00	<0.01	<0.01	<0.01	0.01 ± 0.00	0.09 ± 0.01	0.93 ± 0.02	<0.01	0.17 ± 0.02	0.38 ± 0.01	0.01 ± 0.00	<0.01	0.31 ± 0.05	0.03 ± 0.00	<0.01	1.58 ± 0.21	0.32 ± 0.04	0.12 ± 0.01	0.01 ± 0.00
Carbon	0.05	0.38	4.73 ± 0.31	0.07 ± 0.00	<0.01	<0.01	0.02 ± 0.01	<0.01	<0.01	0.06 ± 0.00	0.49 ± 0.07	<0.01	0.16 ± 0.01	0.12 ± 0.02	<0.01	<0.01	0.05 ± 0.01	0.019 ± 0.00	0.01 ± 0.00	0.5 ± 0.15	0.12 ± 0.03	0.16 ± 0.01	0.06 ± 0.01
	0.31	0.29	2.85 ± 0.17	0.02 ± 0.00	<0.01	<0.01	0.03 ± 0.00	<0.01	<0.01	0.04 ± 0.00	0.23 ± 0.06	0.03 ± 0.00	0.17 ± 0.05	<0.01	<0.01	<0.01	<0.01	0.01 ± 0.00	0.01 ± 0.00	<0.01	0.05 ± 0.01	0.10 ± 0.01	0.04 ± 0.00
Nitrogen	0.05	0.21	4.18 ± 0.25	0.08 ± 0.01	0.01 ± 0.00	0.01 ± 0.00	<0.01	0.05 ± 0.00	<0.01	0.12 ± 0.01	0.25 ± 0.02	<0.01	<0.01	0.12 ± 0.00	0.1 ± 0.01	<0.01	<0.01	0.12 ± 0.02	0.12 ± 0.00	<0.01	<0.01	0.35 ± 0.03	<0.01
	0.31	0.20	2.41 ± 0.16	0.04 ± 0.00	<0.01	<0.01	<0.01	0.04 ± 0.00	<0.01	0.07 ± 0.01	0.17 ± 0.01	<0.01	<0.01	0.04 ± 0.00	0.05 ± 0.00	<0.01	<0.01	0.1 ± 0.01	0.13 ± 0.00	<0.01	<0.01	0.38 ± 0.03	<0.01
Phosphate	0.05	0.66	6.72 ± 0.67	0.14 ± 0.01	0.01 ± 0.00	0.05 ± 0.00	<0.01	0.01 ± 0.01	<0.01	0.02 ± 0.00	0.51 ± 0.06	<0.01	<0.01	3.52 ± 0.56	2.85 ± 0.49	0.05 ± 0.01	6.00 ± 0.14	4.28 ± 0.25	0.25 ± 0.03	100.70 ± 3.24	14.14 ± 0.95	0.07 ± 0.01	<0.01
	0.31	0.53	5.62 ± 0.22	0.09 ± 0.01	0.01 ± 0.00	0.02 ± 0.00	<0.01	0.02 ± 0.01	<0.01	0.02 ± 0.00	0.48 ± 0.03	<0.01	<0.01	1.80 ± 0.61	1.25 ± 0.06	0.02 ± 0.00	3.17 ± 0.32	1.18 ± 0.11	0.13 ± 0.01	38.82 ± 2.02	5.13 ± 0.49	0.14 ± 0.05	<0.01
Iron	0.05	1.29	1.60 ± 0.10	0.02 ± 0.00	<0.01	<0.01	<0.01	<0.01	<0.01	0.01 ± 0.00	0.02 ± 0.00	<0.01	<0.01	0.57 ± 0.06	0.91 ± 0,1	0.07 ± 0.01	1.43 ± 0.19	0.40 ± 0.02	0.01 ± 0.00	7.42 ± 0.35	0.10 ± 0.12	0.35 ± 0.03	0.16 ± 0.01
	0.31	1.02	0.80 ± 0.09	0.01 ± 0.00	<0.01	<0.01	<0.01	<0.01	<0.01	0.01 ± 0.00	0.04 ± 0.00	<0.01	<0.01	0.59 ± 0.12	0.43 ± 0.05	0.02 ± 0.00	1.10 ± 0.13	0.22 ± 0.02	0.01 0.00	1.81 ± 0.16	0.76 ± 0.11	0.48 ± 0.07	0.30 ± 0.02
Magnesium	0.05	1.39	4.85 ± 0.60	0.06 ± 0.01	0.01 ± 0.00	<0.01	<0.01	<0.01	<0.01	0.03 ± 0.00	0.21 ± 0.02	<0.01	<0.01	0.43 ± 0.03	0.31 ± 0.03	0.01 ± 0.00	0.52 ± 0.08	0.15 ± 0.02	0.06. 0.00	0.52 ± 0.1	0.78 ± 004	0.19 ± 0.02	0.05 ± 0.01
	0.31	1.54	1.57 ± 0.18	0.02 ± 0.00	<0.01	<0.01	<0.01	<0.01	0.06 ± 0.01	0.03 ± 0.00	0.39 ± 0.04	<0.01	<0.01	0.34 ± 0.02	0.12 ± 0.02	0.01 ± 0.00	0.19 ± 0.04	0.08 ± 0.01	0.01 ± 0.00	0.11 ± 0.05	0.79 ± 0.05	0.19 ± 0.02	0.04 ± 0.00
Sulphur	0.05	0.30	1.28 ± 0.08	<0.01	<0.01	<0.01	0.02 ± 0.00	<0.01	<0.01	<0.01	0.03 ± 0.01	<0.01	0.08 ± 0.01	2.91 ± 0.13	3.14 ± 0.14	0.01 ± 0.00	3.78 ± 0.16	0.41 ± 0.05	0.23 ± 0.02	122.02 ± 2.11	40.60 ± 1.87	0.29 ± 0.02	0.45 ± 0.03
	0.31	0.17	0.6 ± 0.03	<0.01	<0.01	<0.01	0.05 ± 0.01	<0.01	<0.01	<0.01	0.08 ± 0.04	<0.01	0.12 ± 0.01	0.3 ± 0.07	<0.01	<0.01	0.20 ± 0.06	0.08 ± 0.01	0.02 ± 0.00	4.82 ± 0.2	1.39 ± 0.47	0.39 ± 0.02	0.61 ± 0.03
Anaerobic	0.05	0.18	2.12 ± 0.14	0.15 ± 0.00	0.02 ± 0.00	0.01 ± 0.00	0.30 ± 0.04	<0.01	0.03 ± 0.00	0.14 ± 0.00	0.24 ± 0.01	0.01 ± 0.00	0.03 ± 0.00	0.13 ± 0.07	0.05 ± 0.01	0.01 ± 0.00	0.26 ± 0.03	0.06 ± 0.03	<0.01	0.44 ± 0.01	<0.01	ND	2.42 ± 0.23
	0.31	0.15	0.24 ± 0.02	0.02 ± 0.00	<0.01	0.01 ± 0.00	1.40 ± 0.05	<0.01	<0.01	0.02 ± 0.00	<0.01	0.01 ± 0.00	0.01 ±0.00	0.04 ± 0.00	0.01 ± 0.00	<0.01	<0.01	0.02 ± 0.00	<0.01	0.10 ± 0.00	<0.01	ND	1.75 ± 0.05
Temperature 25°C	0.05	1.02	9.33 ± 0.14	0.14 ± 0.01	0.02 ±0.00	<0.01	0.12 ± 0.06	0.03 ± 0.00	0.02 ± 0.00	0.10 ± 0.01	0.42 ± 0.06	<0.01	0.13 ± 0.02	0.03 ± 0.00	<0.01	<0.01	0.06 ± 0.00	0.02 ± 0.00	<0.01	0.77 ±0.03	0.04 ± 0.00	ND	0.05 ± 0.00
	0.31	0.58	6.04 ± 0.15	0.09 ± 0.01	0.01 ± 0.00	<0.01	0.09 ± 0.02	0.03 ± 0.00	0.02 ± 0.00	0.08 ± 0.01	0.45 ± 0.04	<0.01	0.07 ± 0.01	0.02 ± 0.00	<0.01	<0.01	0.04 ± 0.00	0.02 ± 0.00	<0.01	0.54 ± 0.03	0.04 ± 0.00	ND	0.05 ± 0.00

[a]Quorum sensing signalling molecule production was determined by LC-MS/MS. The mean values are based on six samples taken at steady state. ND, not determined.

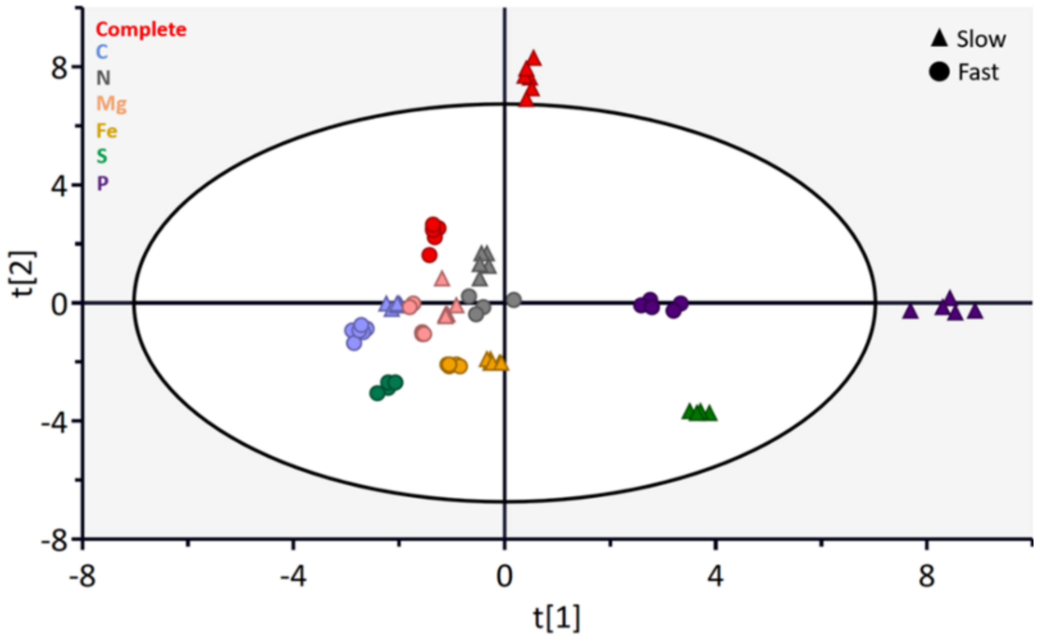

Fig S1. PCA plot of AQ and AHL accumulation in cell free supernatants of *P. aeruginosa* during growth in continuous culture at slow ($\mu = 0.05\ h^{-1}$) and fast ($\mu = 0.31\ h^{-1}$) growth rates with respect to nutrient limitation. AQs and AHLs were extracted and profiled by LC-MS/MS analysis. Data are based on the means of six samples taken at steady state per growth condition and represent variations in QSSM production across the dataset**.**
